# Neurogenic bladder findings in patients with Congenital Zika Syndrome: A novel condition

**DOI:** 10.1371/journal.pone.0193514

**Published:** 2018-03-01

**Authors:** Lucia Maria Costa Monteiro, Glaura Nisya de Oliveira Cruz, Juliana Marin Fontes, Tania Regina Dias Saad Salles, Marcia Cristina Bastos Boechat, Ana Carolina Monteiro, Maria Elizabeth Lopes Moreira

**Affiliations:** 1 Department of Pediatric Urodynamics and Dysfunctional Voiding, Instituto Nacional de Saúde da Mulher, da Criança e do Adolescente Fernandes Figueira (IFF/FIOCRUZ), Rio de Janeiro, Rio de Janeiro, Brazil; 2 Department of Pediatric Neurology, Instituto Nacional de Saúde da Mulher, da Criança e do Adolescente Fernandes Figueira (IFF/FIOCRUZ), Rio de Janeiro, Rio de Janeiro, Brazil; 3 Department of Pediatric Radiology and Imagining, Instituto Nacional de Saúde da Mulher, da Criança e do Adolescente Fernandes Figueira (IFF/FIOCRUZ), Rio de Janeiro, Rio de Janeiro, Brazil; 4 Department of Internal Medicine, University of California Los Angeles, (U.C.L.A), Los Angeles, California, United States of America; 5 Department of Neonatology, Instituto Nacional de Saúde da Mulher, da Criança e do Adolescente Fernandes Figueira (IFF/FIOCRUZ), Rio de Janeiro, Rio de Janeiro, Brazil; The Cleveland Clinic, UNITED STATES

## Abstract

**Introduction:**

Congenital Zika Syndrome (CZS) has been associated with microcephaly and other central nervous system abnormalities including areas that have been implicated in the control of the lower urinary tract. As such, this descriptive case series has aimed to investigate whether CZS is linked with neurogenic bladder. Identifying such an association is paramount in the effort to recognize CZS complications that have putative treatment options that could mitigate the impact of CZS in infected children.

**Methods:**

Following IRB approval, urological assessment was performed in all patients referred to our clinic between June 2016 and May 2017 who presented with confirmed CZS-associated microcephaly. The research protocol consisted of obtaining clinical history, laboratory tests, lower and upper urinary tract ultrasounds, as well as a diagnostic urodynamic evaluation. ZIKA virus infection was previously confirmed by maternal history and positive PCR in babies and mothers. Microcephaly and other central nervous system abnormalities were established based on neurological assessment and associated imaging of the central nervous system (CT head and/or Brain MRI).

**Results:**

Twenty-two consecutive CZS patients were tested and confirmed to have neurogenic bladder. Of the 22 patients assessed, 21 presented with an overactive bladder combined with reduced bladder capacity and elevated detrusor filling pressures. Clinically significant increases in postvoid residual (PVR) were confirmed in 40% of cases while a urinary tract infection (UTI) was identified in 23% of cases.

**Conclusion:**

Neurogenic bladder, a known treatable health condition, was confirmed in 100% of patients tested in this study, most presenting with high-risk urodynamic patterns known to lead to renal damage when left untreated. Follow up studies are necessary to provide further insight onto long-term disease progression and to investigate the response to standard therapies for neurogenic bladder. Nonetheless, we emphasize the importance of proactive management of neurogenic bladder and prompt referral so as to help mitigate CZS disease burden for patients and their families.

## Introduction

Congenital Zika Syndrome (CZS), as initially identified in 2015 in Brazil, continues to challenge neonatologists and pediatricians worldwide as studies continue to support the association of congenital abnormalities in babies to mothers exposed to Zika virus (ZIKv) [1][2][3][4][5]. CZS includes a spectrum of neurological abnormalities ranging from minor injuries in neurodevelopment to microcephaly. Severe cerebral damage has been consistently confirmed and characterized by brain imaging of children affected by CZS [6][7][8]. Current studies are underway to further investigate the later sequelae of the disease in postnatal development and its effects on end organ dysfunction. We now focus on investigating the myriad of possible functional repercussions to end organ systems. Importantly, we recognized that Zika virus affects many of the structures of the central nervous system (CNS) that control the lower urinary tract. As such we suspected that Zika-related CNS effects could also result in neurogenic bladder.

Congenital neurogenic bladder is a well-know treatable health condition [9] and its proactive management reduces the progression to end-stage renal disease [10]. However, if not intervened upon in a timely manner, neurogenic bladder can cause progressive urinary system damage, advancing to chronic stages which may involve lifelong burden of recurrent urinary tract infections, urinary incontinence and ultimately chronic and end-stage renal disease. Such complications impose a high resource burden on the healthcare system, as it may demand recurrent hospitalizations, dialysis dependence and need for organ transplantation. Unlike most outcomes in the setting of CZS, the upper urinary tract complications described may be prevented with pre-established therapies. However, the essential initial step in preventing these complications is to encourage primary care, neurology and infectious disease providers to promptly refer CZS patients who may have urinary dysfunction to the urology clinic.

As such, this study aims to investigate a possible relationship between Congenital Zika Syndrome and neurogenic bladder

## Methods

This is a descriptive case series study including all pediatric patients with confirmed microcephaly due to CZS who were referred for urological assessment between June 2016 and May 2017. The patients in this cohort came from the CZS clinics at The Fernandes Figueira Institute/FIOCRUZ, a Ministry of Health referral center for high-risk pregnancies and pediatric infectious diseases in Rio de Janeiro, Brazil. The CZS cohort clinics are currently following 303 vertically exposed children, including 63 babies with microcephaly.

Diagnosis of ZIKv was confirmed by maternal history (rash during pregnancy and/or low fever, myalgia, arthralgia, fatigue, conjunctival hyperemia) and positive PCR in mothers and/or babies. The presence of CNS abnormalities was identified based on reports of neurological evaluation and findings of transfontanelle doppler ultrasound, brain imaging CT and MRI. Microcephaly was diagnosed based on a measured head circumference that was 2 standard deviations below the mean for gestational age and sex [11].

Urological evaluation was based on the protocol to investigate neurogenic urological disorders in children [12], in agreement with the International Children’s Continence Society [9]. This protocol included a clinical history, laboratory tests, renal and bladder ultrasounds (US) and a diagnostic urodynamic study (UDS). The clinical history focused on whether the child had urinary continence as well as the number of episodes of urinary tract infection (UTI) that had been confirmed by urinalysis and culture. UDS was performed after ruling out UTI. Our clinic specializes in pediatric urodynamics and all professionals are trained to assist patients with special needs. The parents and the child were familiarized with the urodynamic procedure and catheterization during a clinic visit that was scheduled one week before the exam. The examination room is adapted to promote a child-friendly environment, which includes toys for various age groups, as well as video entertainment showcasing a variety of shows and movies catered to specific age groups. Both parents are encouraged to stay in the room during the entire procedure so as to comfort and support the child. Such interventions have enabled us to perform Urodynamic tests without the need for general anesthesia or dissociative medications. The urodynamic tests performed were a cistometrogram (CMG) and an electromyography (EMG). The CMG was performed after urethral catheterization with a 6Fr double lumen pediatric catheter, under topical anesthesia with lidocaine gel (1%). The urinary residual volume was measured after taking into account markers of recent voiding such as diaper wetness and/or voiding before or during catheterization. The urinary residual volume at the beginning of the exam was compared to the post-void/residual volume at the end of the test. The filling rate was then calculated based on the expected bladder capacity for age (ml/min). In general, patients with neurogenic bladder do not express a strong urge to urinate. Bladder capacity was measured just before the child leaked or voided, or when the child displayed discomfort, when bladder baseline pressures stayed above 40 cm H_2_O or when the volume infused was 1.5 times the expected capacity for the patient’s age. The maximum bladder pressure was measured at the bladder capacity. The leak point pressure was measured during the first leak and the voiding pressure was measured when more sustained emptying was observed. Detrusor overactivity was defined by the presence of involuntary detrusor contraction above 15 cm H_2_O from baseline during bladder filling. Whenever possible, pressure measurements were made considering the baseline and not bladder contraction. Electromyography was performed using patch EMG electrodes. The sphincter function was considered normal (detrusor sphincter synergia) when sphincter activity remained present during the filling phase and was noted to be reduced following a high bladder pressure during contraction, or during a leak or a void. Detrusor sphincter dyssynergia (DSD) was considered when the sphincter failed to relax, or increased its activity following a high bladder pressure during contraction, or during a leak or a void [13]. A urodynamic diagnosis was established based on bladder and urethral sphincter behavior during filling and voiding phases, considering:

- detrusor and sphincter activity,
- maximum bladder pressure and micturition/leak point pressure,
- bladder capacity [14] and bladder compliance.

The research was approved by the Research Ethics Committee of Instituto Fernandes Figueira—IFF/FIOCRUZ—RJ/ MS under registration CAAE -60168616.4.0000.5269. The CZS cohort is registered in ClinicalTrials.gov (NCT03255369). Parental written informed consent was required for the inclusion of the patient into the study. Parents of patients discussed in this manuscript have given written informed consent to publish the details of this study.

## Results

In one year, a total of 23 patients with CZS were referred for urological assessment in our clinic. All 23 patients referred had imaging-confirmed central nervous system malformations, either from Brain CT or MRI. Of the 23 referred patients, one was excluded from the study as he was also diagnosed with myelomeningocele (MMC), a well documented cause of neurogenic bladder and a potential confounder for this investigation.

From the 22 patients included (S1 File), 14 were female and and 8 male, their age at admission ranged from 3 to 16 months with a mean of 9.8 months. All patients were diaper dependent, as is expected for their age group, and urinary incontinence was difficult to evaluate.

The urological assessment for the cohort is presented in Table 1. Urinary tract infection was confirmed in 5 out of 22 patients. Renal US was abnormal in 2 out of 22 patients- one patient had multicystic dysplastic kidney disease (MCKD) and contralateral discrete renal pelvis dilatation, and the other had a kidney stone. No structural alteration to the bladder was observed in any of the patients. One patient had a bladder US revealing urinary debris and a UTI was later confirmed. The patient with pelvic dilatation had a normal renal scan, and as such vesicoureteral reflux (VUR) is being investigated, but has not yet been confirmed. All 22 patients in the cohort had urodynamic tests confirming neurogenic bladder, 21 of which presented with overactive bladder, one of them with detrusor sphincter dyssynergia. One patient presented with a (likely underactive) relaxed bladder during the filling phase, with a continuous rise in the bladder pressure starting at 150% of the expected bladder capacity for age. Her leak point pressure was 50 cm H_2_O and her void was incomplete, resulting in a post-void residual of 100 ml, compared to the 18 ml collected after catheterization at the beginning of the exam. She arrived with a very wet diaper. Given the hypotonicity of her bladder far beyond that observed in the rest of the cohort, a confounding cause was suspected. Although all patients were in use of anticonvulsant medication for seizure control, she was the only one who was lethargic during the UDS. As such, this patient was referred for neurological re-evaluation and which revealed that her neurological condition had deteriorated.

**Table 1:** Urological assessment in 22 consecutive patients with confirmed Congenital Zika Syndrome.

		N (%)
**Urinary Culture (N = 22)**		
	Normal	17 (77.27%)
	Urinary Tract Infection (UTI)	5 (22.73%)
**Renal Ultrasonography (N = 22)**		
	Structurally Normal Urinary System	20 (90.91%)
	Renal abnormalities	2 (9.09%)
	Bladder abnormalities	0
**Urodynamic studies (N = 22)**		
**Bladder:**	Normal	0
	Overactive	21 (95.45%)
	Relaxed (probably underactive)	1 (4.55%)
**Urethral Sphincter (N = 22)**	Detrusor sphincter synergia	21 (95.45%)
	Detrusor sphincter dyssynergia	1 (4.55%)

Different urodynamic patterns of overactive bladder were observed, but the majority of patients examined presented with reduced bladder capacity and abnormally high detrusor filling pressure, both well established high-risk urodynamic indicators that frequently cause progressive urinary system damage if left untreated [15][16]. Of note, it is well documented that the risk for upper urinary tract compromise is related to the length of time that the bladder maintains a high detrusor pressure during the filling phase [13][17]. Our preliminary assessment suggests that CZS patients have prolonged periods of high detrusor pressure.

**Fig 1:**
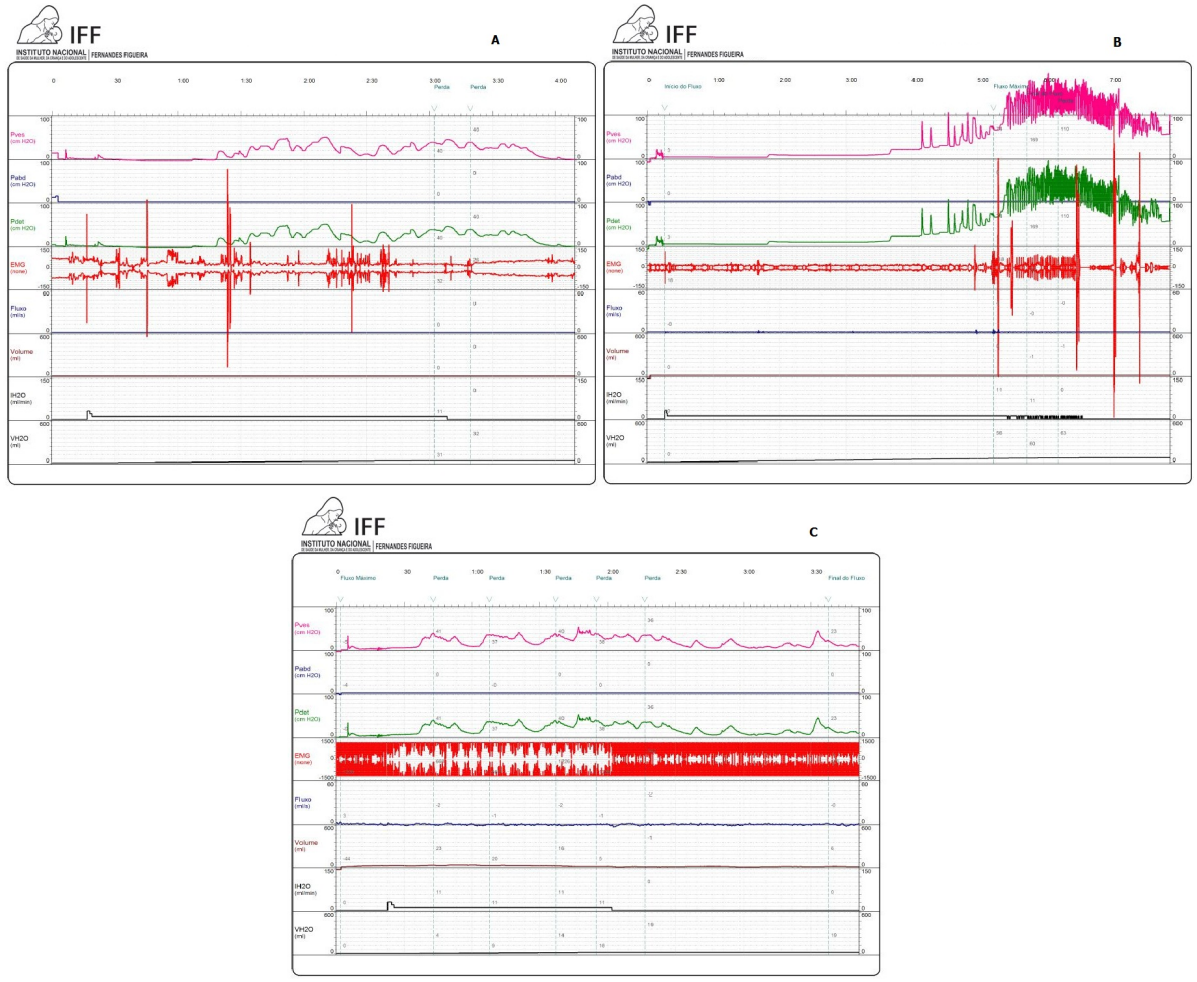
Urodynamic studies found on CZS patients. Urodynamic studies showing three different scenarios of overactive bladder found on CZS patients, all with high-risk urodynamic indicators known to cause progressive urinary system damage. A (case 1): Bladder behavior is normal at the beginning but a series of uninhibited detrusor contractions raises the bladder pressure during 2/3 of the filing phase. B (case 2): A very high and sustained inhibited detrusor contraction and a concomitant increased sphincter activity (detrusor-sphincter dyssynergia) raises the intravesical pressure up to 100 cm H_2_0. The leak point pressure is equally dangerously high (110 cm H_2_0). C (case 3): The repeated inhibited detrusor contractions starting at the very beginning of the filing phase, always followed by leak, severely reduces the bladder capacity.

A variety of urodynamic profiles were observed in this study. To illustrate a cross section of observed patterns, three examinations have been included here. Fig 1A shows the CMG exam of a patient that reveals a normal baseline pressure for the first 1/3 of the filling phase, followed by a series of inhibited detrusor contractions with a maximum detrusor pressure of 42 cm H_2_O. The detrusor pressure drops to 20 cm H_2_O when the contractile phase ends. This patient’s first urinary leak occurred at 40 cm H_2_0, when the sphincter relaxed at peak pressures and the contraction was sustained to complete micturition. A different urodynamic scenario is presented in Fig 1B. The patient’s baseline pressure was normal for the first 2 minutes of the filling phase, when the detrusor pressure started to rise steadily and progressively, without returning to baseline until the sphincter relaxed and the patient voided with a pressure of 110 cm H_2_O. A high detrusor pressure was observed during most of the filling phase, greatly raising the risk of kidney damage. The third patient (Fig 1C) had a CMG exam that revealed repeated inhibited detrusor contraction starting at the beginning of the filling phase, followed by a series of sphincter relaxations and small urinary leaks at around 30 cm H_2_O, which reduced the observed bladder capacity. All three patients described were treated with anticholinergic drugs. Clean intermittent catheterization (CIC) was also recommended to the patient whose exam was described in Fig 1B.

The summary of urodynamic findings for all 22 patients is presented in Table 2, confirming an average high maximum detrusor pressure and high leak point pressure. The average bladder compliance and the bladder capacity were very low compared to the general age adjusted population. Importantly, the findings described are all high-risk urodynamic indicators for renal impairment. All patients noted to have overactive bladder were treated with anticholinergics. Patients were also recommended to have scheduled clean intermittent catheterization whenever their post-void residual was consistently higher than 20% of the bladder capacity or their baseline detrusor pressure was kept above 40 cm H_2_O. All parents were instructed to observe the number and the weight of the diapers changed per day and take note of any change in the color and/or smell of the patient’s urine.

**Table 2:** Urodynamic parameters measured in 22 consecutive patients with Congenital Zika Syndrome and microcephaly.

	Maximum	Minimum	Median	Mean
_Max Bladder Pressure (cm H_2_O)_	200	35	63	81.1
_Leak Point Pressure (cm H_2_O)_	202	22	53	73.0
_Bladder Compliance (ml/cm H_2_O)_	3.44	0.05	0.47	0.69
ratio between Maximum and Age Adjusted Expected bladder capacity	2.46	0.08	0.39	0.65

The ability to void effectively is a crucial indicator of lower urinary function. The post-void residual was measured at the beginning of the study, just after catheterization and at the end of the study, after voiding or leaking. PVR was evaluated based on the percentage of the maximum bladder capacity measured during CMG. Results are presented in Table 3. All patients but one arrived with a wet diaper, and 18 managed to void at the end of the CMG. Ten voided effectively (PVR = 0). These patients were treated with anticholinergic drugs for overactive bladder and parents were instructed to count wet diapers per day and time between voiding events. Nine patients had a PVR above 50% of bladder capacity at the end of the CMG; of those, 5 also voided ineffectively at the beginning of the exam. Regardless of US results, these patients are being further investigated for VUR and had CIC included in their treatment schedule, combined with anticholinergic drugs for overactive bladder. All patients will continue to be monitored according to established protocol [12].

**Table 3:** Post-void residual measured during urodynamic study in 22 consecutive patients with Congenital Zika Syndrome and microcephaly.

Voiding before and after CMG (n = 22)	Before CMG	After CMG
wet diapers on arrival	21	-
voiding	-	18
leak without voiding	-	4
Post Void residual (PVR)		
no PVR (voiding was complete)	9	10
PVR between 1%–20% of Bladder Capacity	2	0
PVR between 21%–50% of Bladder Capacity	6	3
PVR above 50% of Bladder Capacity	5	9

## Discussion

Modelling studies have predicted the overwhelming risk of the global spread of Zika virus[18][19], with potential involvement of over 65 countries and subsequent staggering societal costs. Reports estimated a financial burden of $3.5 billion dollars in Latin America alone in 2016 [20]. Notably, the number of pregnant women with laboratory evidence of Zika virus infection and the number of fetuses/infants with Zika virus–associated birth defects continues to increase. In the United States, the proportion of fetuses and infants with birth defects among pregnancies with confirmed Zika virus infection at any time during pregnancy is more than 30 times higher than the baseline prevalence in the pre-Zika years [21]. Health care for Zika-infected infants over their lifetimes will exceed hundreds of billions of dollars[20] if the health-related morbidities are not prevented and/or mitigated.

The affected areas of cerebral damage found in the imaging studies of the patients in our series were similar to the ones found in the majority of patients with Congenital Zika Syndrome [22]. They also involved the brain regions associated with neurological control of the lower urinary tract [23]. Neurogenic bladder was confirmed in all patients tested so far. This is the first published report of neurogenic bladder as a sequela of Congenital Zika Syndrome.

Neurogenic bladder happens when an injury to the nervous system reaches the micturition centers and disrupts the physiological functioning of the bladder and urinary sphincter. This compromises the physiology and hydrodynamic flow of the urinary system, causing urological dysfunction of varying degrees, which affects kidney function and may cause renal failure if not properly diagnosed and treated[24]. Urodynamic studies have become the gold standard for diagnosing neurogenic bladder [14] because it identifies pathophysiological risk factors that lead to upper urinary tract damage.

Treatment strategies for neurogenic bladder from any cause have aimed to protect the upper urinary tract, ensuring a bladder with appropriate capacity and compliance that effectively empties. Standard therapy includes anticholinergic drugs to control overactive bladder and low bladder compliance, intermittent clean catheterization (CIC) whenever bladder emptying is ineffective, and antibiotics for treatment of confirmed urinary tract infections [12]. Conversely, abnormal urodynamic findings such as low bladder capacity, low bladder compliance, bladder overactivity with consistent high detrusor pressure during filling phase and detrusor-sphincter dyssynergia are well known high-risk urological indicators that can damage the upper urinary tract. All 22 CZS patients presented with abnormal urodynamics. Moreover, results from urodynamic tests in most of the subjects revealed high-risk urological indicators that may lead to renal impairment. It is important to highlight that even when high risk indicators are identified, early intervention in neurogenic bladder can reverse risk factors. As such, this report recognizes an end organ condition associated with CZS that is treatable and reversible, with potential for significantly mitigating the burden of this disease in the affected population.

Abdominal US, including renal assessment, was part of the initial screening protocol for the Zika cohort studies and was normal in the majority of patients. We learned from experience with patients diagnosed with myelomeningocele, to date the most common cause of congenital neurogenic bladder, that the renal US is expected to be normal in newborns but, when high-risk urological indicators are present, urological deterioration is expected to develop after birth and during the first year of life [25]. When the baseline bladder pressure continuously exceeds 40 cm H_2_O during the filling phase, the upper urinary tract function will be at risk of being impaired [26]. The urodynamic patterns presented here confirm one or more urological risk factors in most patients tested, increasing their risk for future renal dysfunction. The average maximum bladder pressure measured in these studies was twice as high as normal range. In addition, the average bladder capacity and compliance were incredibly low in the majority of cases, and these are also very important indicators of urinary tract ill-health.

Of concern, half of CZS patients assessed had a significant post void residual at the end of the CMG, even with a synergic sphincter and a complete void in the diapers before the exam. Renal and bladder US were observed to be normal in most of the cases and VUR was not confirmed so far. This needs to be further investigated, ideally with video-urodynamic studies if available, to better evaluate if VUR occurs during increased detrusor pressures, urinary leakage or voiding, and to better evaluate bladder neck and sphincter behavior during the filling and voiding phases.

One of the limitations of our study is the absence of a control group. The ideal control would be the children from mothers infected with Zika virus during their pregnancy born without microcephaly or CNS sequela. However, as the urodynamic test is an invasive procedure, it was considered unethical to test asymptomatic patients. An alternate approach would be to create a clinical control group, based on other neurogenic bladder diagnoses. However, we have noticed that the urological patterns of Zika patients are far too different from other patients with neurogenic bladder that we have followed for the last 25 years [15] in a way that would make any comparison unrealistic.

CZS is a new disease that affects multiple organ systems and brings a wide spectrum of neurological abnormalities for affected babies. Neurogenic bladder was confirmed in 100% of patients tested in this study. Starting treatment of neurogenic bladder within the first year of life triples the probability of urodynamic improvement in patients with spina bifida, thereby reducing the risk of upper urinary tract damage [15]. It is fair to believe that the CZS patients will also benefit from this preventive approach.

Conversely, it is known that untreated congenital neurogenic bladder advances to chronic stages and cause progressive damage to the urinary system, increasing morbidity and raising treatment costs. Delayed diagnosis and treatment is the central risk factor leading to worse outcomes. However, referrals are made based upon observable symptoms, and unfortunately urological symptoms tend to be silent or misinterpreted in neonates and toddlers. Urinary tract infection (UTI), caused by urinary retention and bladder wall ischemia due to high bladder pressure, is a frequent symptom in neurogenic bladder. But it remains one of most difficult conditions to diagnose, treat and prevent in neonates. In the study group, a UTI was confirmed only in 5 of 22 of patients tested. Frequent UTIs also increase the risk of nephropathy [27]. Urinary incontinence (UI) is another frequently associated symptom that imposes a great burden to patient’s health and quality of life that will be unnoticed in this age group. It is mostly caused by low bladder capacity and overactive bladder, which were diagnosed in most of the CZS patients. Equally, we believe that failure to recognize and treat these conditions in CZS patients may also lead to lifelong incontinence, a central burden to patients and families.

Similarly to other causes of congenital neurogenic bladder, parents of CZS patients are burdened with a demanding multidisciplinary clinical agenda. The lack of urological symptoms at this early stage comes in conflict with the parents’ and clinicians’ urge to treat and investigate other more visible sequelae of CZS. This is contributing to a costly delay in urodynamic investigation, loss to follow-up and decreased treatment adherence.

Prompt diagnosis and proactive management of neurogenic bladder within the first year of life will prevent UTIs, urinary incontinence and deterioration of renal function [15][28][29][30], but we fear that not all patients in the settings of CZS are being evaluated. Based on our results, we recommend preventive urological referral to all CZS patients with microcephaly and other central nervous system abnormalities. There is a very narrow window of opportunity for preventive and proactive management of congenital neurogenic bladder, but CZS children are getting older and losing the opportunity for treatment. We hope that by sharing our study results and the importance of proactive management, we can increase awareness regarding the need for further urological assessment in these patients.

## Conclusions

Neurogenic bladder, a treatable health condition, was confirmed in 100% of patients tested in this study, most with high-risk urodynamic patterns that may cause renal damage if left untreated. While further investigation is necessary to understand long-term disease behavior and therapeutic response in the setting of Congenital Zika Syndrome, the proactive management of neurogenic bladder may help mitigate disease burden for patients and their families.

## Supporting information

S1 File This is the excel spreadsheet containing research data. (XLSX)
